# Infection dynamics of *Salmonella* Infantis strains displaying different genetic backgrounds – with or without pESI-like plasmid – vary considerably

**DOI:** 10.1080/22221751.2021.1951124

**Published:** 2021-07-22

**Authors:** Victoria Drauch, Christian Kornschober, Nicola Palmieri, Michael Hess, Claudia Hess

**Affiliations:** a Clinic for Poultry and Fish Medicine, Department for Farm Animals and Veterinary Public Health, University of Veterinary Medicine, Vienna, Austria; b National Reference Centre for Salmonella, AGES, Graz, Austria

**Keywords:** *Salmonella* Infantis, broiler chicken, pESI-like plasmid, virulence, infection, experimental study

## Abstract

Food-borne infections with *Salmonella* are among the most common causes of human diseases worldwide, and infections with the serovar Infantis are becoming increasingly important. So far, diverse phenotypes and genotypes of *S*. Infantis have been reported. Therefore, the present study aimed to investigate the infection dynamics of two different *S*. Infantis strains in broilers. For this purpose, 15 birds were infected on day 2 of life with 10^8^ CFU/ml of a pESI+ or a pESI– *S*. Infantis strain, respectively. Ten uninfected birds served as in-contact birds to monitor transmission. In both groups, an increase of infection was observed from 7 days of age onwards, reaching its peak at 28 days. However, the pESI+ strain proved significantly more virulent being re-isolated from most cloacal swabs and organs by direct plating. In contrast, the pESI– strain could be re-isolated from cloacal swabs and caeca only when enrichment was applied. Although the excretion of this strain was limited, the transmission level to in-contact birds was similar to the pESI+ strain. Differences in infection dynamics were also reflected in the antibody response: whereas the pESI+ strain provoked a significant increase in antibodies, antibody levels following infection with the pESI– strain remained in the range of negative control birds. The actual findings provide for the first time evidence of *S*. Infantis strain-specific infectivity in broilers and confirm previous observations in the field regarding differences in persistence on farms and resistance against disinfectants.

## Introduction

Worldwide, *S*. Infantis is actually reported as the most common isolated serovar from animal and food sources, with the majority of strains originating from broilers [1–6]. In consequence, over the last years, this serovar has also become a relevant agent of human salmonellosis [2, 7–9]. Interestingly, recent genomic studies revealed that the population of *S*. Infantis is heterogeneous and consists of different clones and clusters [10–12]. These differences are also evident in the antibiotic resistance profiles being mainly represented by either pan-susceptible or multidrug resistant strains [4, 10, 13, 14]. Multidrug resistant *S*. Infantis strains were frequently found to be associated with conjugative pESI-like megaplasmid are of global concern for the poultry industry as well as human medicine [15–20]. An increased fitness under various environmental conditions is reported for *S*. Infantis strains with difficulties of elimination from farms or slaughterhouses, despite extensive cleaning and disinfection [21–23]. In this respect, we could recently demonstrate that *S*. Infantis strains which persist on farms were more resistant to disinfectants [24].

*In vivo* infection experiments in mice demonstrated a higher pathogenicity and more inflammatory reactions by *S*. Infantis strains with multidrug resistance (MDR) [15]. So far, experimental infections in chickens mainly focused on layer-type birds, and/or investigated a single *S*. Infantis strain [25–27]. By this, actually, there are no data available regarding the genetic diversity of strains and their influence on the infection dynamics in broiler birds, the main host of these bacteria.

Therefore, the present study focused on the colonization behaviour and antibody response of two different *S.* Infantis strains in commercial broilers. For this purpose, the bacterial load in organs and faecal shedding were determined after infection altogether completed by using in-contact birds to unravel infection dynamics.

## Material and methods

### Bacteria

Two phenotypically different *S.* Infantis field strains, MRS-16/01939 and MRS-17/00712 derived from Austrian broiler flocks, were used. Whereas strain MRS-16/01939 proved multidrug resistant and showed growth of black colonies on xylose–lysine–deoxycholate agar (XLD, Merck, Vienna, Austria), strain MRS-17/00712 was pan-susceptible presenting colourless colonies on XLD. Furthermore, strain MRS-16/01939 was reported to persist on the farm leading to recurrent infections, whereas strain MRS-17/00712 was only isolated once from the farm.

### Sequencing and analysis

Genomic DNA was isolated from overnight cultures using the MagAttract HMW DNA Kit (Qiagen, Hilden, Germany). Paired-end sequencing was performed on a MiSeq platform (Illumina Inc., San Diego, CA, USA). Library preparation was carried out using Nextera XT according to the instructions of the manufacturer (Illumina Inc., San Diego, CA, USA). Raw reads were quality controlled using FastQC v0.11.9. For assembly, raw reads were de novo assembled using SPAdes version 3.11.1 (St. Petersburg State University, Center for Algorithmic Biotechnology, RU) [28]. Whole-genome microbial typing was based on the EnteroBase cgMLST scheme for *S*. enterica (M. Achtmann, Warwick, UK) integrated in the SeqSphere+ Software version 7.2 (Ridom, Münster, Germany) [29].

For phylogenetic analysis, both *S*. Infantis strains were first analysed through the Salmonella *In Silico* Typing Resource (SISTR) online tool [30], to determine the two closest strains. Then, all 45 complete genomes of *S*. enterica serovar Infantis were downloaded from NCBI (08-04-2021) and employed for tree construction using parsnp (default parameters – correcting for recombination). Screening for the presence of pESI-like megaplasmid structures was performed by aligning the pESI plasmid from strain 119944 (accession: CP047882.1) to the assemblies of MRS-16/01939 and MRS-17/00712 using the module Whole Genome Alignment from CLC Genomics Workbench version 21.0.3 [31].

### Birds and housing

The animal trial was approved by the institutional ethics committee and the national authority according to section 8ff of the Law for Animal Experiments, Tierversuchsgesetz (license number GZ.: 68.205/0157-V/3b/2019).

A total of 75 one-day-old ROSS 308 broilers (Brueterei Schulz, Lassnitzhoehe, Austria) were divided into 3 groups with 25 birds each. Birds were subcutaneously marked with an individual number tag (Swiftag^™^, Heartland Animal Health Inc. Fair Play, MO, USA) and each group was housed separately in isolators (Montair HM2500, Montair Environmental Solutions B.V., Kronenberg, The Netherlands). Feed and water were provided *ad libitum*.

### Preparation of inoculum

*Salmonella* Infantis strains were grown on MacConkey agar (Bertoni, Vienna, Austria) at 37°C for 24 h. Selected fresh colonies were inoculated in Luria–Bertani broth (LB, Invitrogen, Vienna, Austria) at 37°C overnight with agitation (250 rpm). The concentrations of bacteria in the inoculums were determined by colony-forming unit (CFU) counts on MacConkey agar in serial dilutions (1:10) in duplicate. The bacterial suspensions were washed and re-suspended in phosphate-buffered saline (PBS, GIBCO, Paisley, UK). The inoculation dose was 6 × 10^8^ CFU/ml for MRS-16/01939 and 3 × 10^8^ CFU/ml for MRS-17/00712.

### Experimental design and sample collection

At the second day of life, 15 birds each from groups 1 and 2 were orally infected with 1 ml of *S.* Infantis strain MRS-16/01939 or MRS-17/00712, respectively. Oral application was performed by using a crop tube attached to a syringe. The remaining 10 birds in each group stayed non-infected and served as in-contact birds. Birds of group 3 remained uninfected as a negative control with 1 ml of PBS applied orally as described above.

Birds were investigated daily for clinical symptoms together with the housing conditions (temperature, humidity, air pressure, and airflow). Before, and weekly after the experimental infection two cloacal swabs (Copan, Stoelzle-Oberglas GmbH, Vienna, Austria) per bird and blood samples from all birds were collected (*V. basilica*). Following experimental infection 5 birds, 3 infected, and 2 in-contacts from groups 1 and 2, together with 5 birds from the negative control group were euthanized at 7, 14, 21, 28, and 35 days of age by injecting a mixture (1:1) of Narketan^®^ (100 mg/ml, Vetoquinol, Vienna, Austria) and Sedaxylan^®^ (20 mg/ml, Dechra Pharmaceuticals, Dornbirn, Austria) intramuscularly (breast muscle) with subsequent bleeding out by cutting the *V. jugularis*. Necropsy was performed according to a standard protocol. Body weight, weight of liver and spleen, and gross pathological lesions were recorded. Tissue samples from liver, spleen, and caecum were collected for bacteriology.

### Bacteriology

Weekly taken pairwise cloacal swabs were investigated to determine the shedding behaviour of both strains. For this purpose, one cloacal swab was directly streaked on XLD and brilliant-green phenol-red lactose sucrose agar (BPLS, Bertoni, Vienna, Austria). Both cultures were incubated at 37°C for 24 h. The second swab was stored in the fridge (4–8°C) until the result of direct plating was available. In case of a negative result, the second swab was investigated by an enrichment procedure according to EN ISO 6579-1:2017 [32]. Shedding of *S*. Infantis was defined as: (a) negative: no growth by direct plating and enrichment, (b) low shedding: growth only by enrichment, and (c) high shedding: growth by direct plating.

For bacterial quantification from organs, 1 g of liver, spleen, and caeca samples were homogenized in PBS (ULTRA TURRAX T 10 basic, IKA, Staufen, Germany), and plated on XLD and BPLS agars in 1:10 dilutions in duplicates. Following incubation of plates at 37°C for 24 h, colonies were counted, and the mean value was calculated per organ sample. The bacterial loads of *S*. Infantis were expressed as CFU/g. Additionally, organ suspensions were stored in the fridge (4–8°C) until results from CFU counts were available. Negative tissue samples by direct plating were processed by applying the enrichment method as described for cloacal swabs.

### Serology

Antibody response against MRS-16/01939 and MRS-17/00712, used as antigens, in two separate indirect ELISAs, was determined based upon an in-house protocol [33]. Briefly, sera were collected prior to infection and in weekly intervals from all birds of groups 1, 2, and 3. Bacterial cells were washed, and 96-well ELISA plates (Nunc Medisorb; Thermo Scientific, Roskilde, Denmark) were coated with each strain separately. Plates were dried at 52°C, and wells were treated with 200 µl blocking buffer (StartingBlock^™^ PBS Blocking Buffer, Thermo Fisher Scientific, Vienna, Austria) for 1 h. After aspiration of the blocking solution, 100 µl of test sera (diluted 1:500) were pipetted into each well in duplicates and incubated for 1 h. After washing, 100 µl of 1:5000 diluted Goat-Anti Chicken IgY (H + L)-HRP (Southern Biotechnology, Birmingham, AL, USA) was added to each well, and plates were again incubated for 1 h at room temperature. Following a second washing step, 100 µl of tetramethylbenzidine substrate (Calbiochem, Darmstadt, Germany) was added to each well, and plates were placed in the dark. After 12 min of incubation, colour reaction was stopped by adding 100 µl of 0.5 M H_2_SO_4_ to each well, and the optical density (OD) was measured with an ELISA reader (Sunrise-Basic; Tecan, Groedig, Austria) at a wavelength of 450 nm. Samples were run in duplicates on both ELISAs to calculate and analyse the mean OD values.

### Statistical analysis

Data were analysed in R Core Team [34] with the use of three packages (nortest, ggplot2 and ggpubr). In a first step, Exploratory Data Analysis was applied to each dataset, and normality was assessed by the Anderson–Darling test. In the case of normal distribution of data, the *t*-test was performed to determine significant differences. When normal distribution of data was not present, Wilcoxon–Mann–Whitney test and Kruskal–Wallis test were carried out. Descriptive analyses were performed for data for which the above-mentioned tests could not be applied.

## Results

### Sequencing and analysis

**Figure 1.**
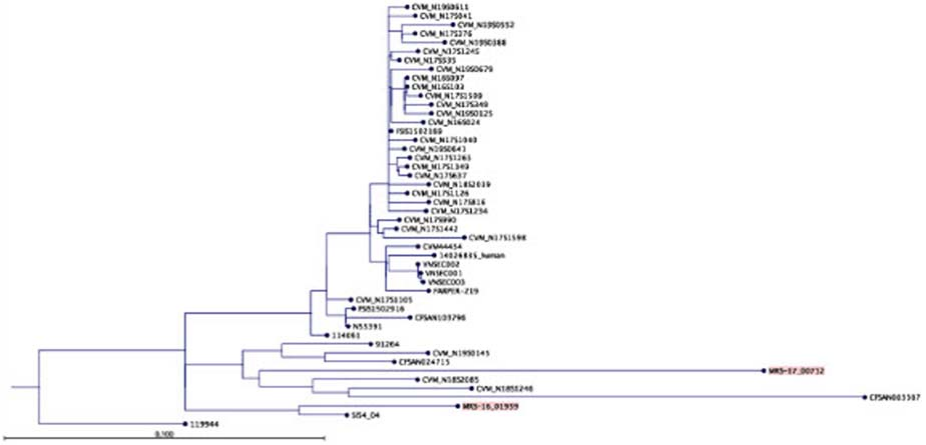
Phylogenetic tree showing the position of S. Infantis strains MRS-16/01939 and MRS-17/00712 compared to other S. Infantis strains (see “Methods” section for details of strains chosen for tree reconstruction).

**Figure 2.**
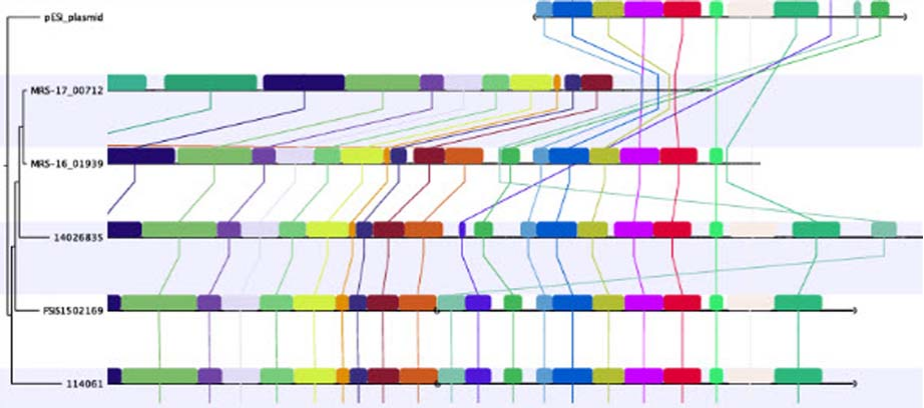
Genomic alignments of the pESI plasmid among S. Infantis strains MRS-16/01939 and MRS-17/00712, the reference strain 114061 and strains 14026835 and FSIS150219. Orthologous blocks are depicted in the same colour and connected by vertical lines.

Applying whole-genome sequencing, both strains could be assigned to the sequence type 32, but they grouped into different genetic cluster types: strain MRS-16/01939 to cluster type 882, and strain MRS-17/00712 to cluster type 906. Phylogenetic analysis revealed that the strains are clearly genetically different from each other (Figure 1), displaying a total of 298 SNPs between their genomic sequences. No difference in chromosomal virulence genes between both strains was found. But, when comparing the assemblies with other published strains, the presence of a pESI-like plasmid was revealed in strain MRS-16/01939 (Figure 2). The genes *irp2, ipf, klf*, and *ccdB/ccdA* known to be located on this plasmid were present. Furthermore, two plasmid-encoded fimbrial operons, *pef* and *sta*, were also detected. The sequences were submitted to the NCBI database under accession numbers SAMN19328299 and SAMN19328300. Based on such differences, strain MRS-16/01939 will be referred to as pESI+ strain and MRS-17/00712 as pESI– strain throughout the manuscript.

### Clinical symptoms, body weight, and gross pathological lesions

No clinical symptoms were recorded during the whole experimental study. No statistically significant differences were observed in body weight, liver, or spleen weight of the birds infected or the negative control (*p* > 0.1). Furthermore, no gross pathological lesions were seen in any of the birds, except for one bird infected with pESI+ strain, which died at 11 days of age due to yolk sac infection. This bird was not included in further analysis resulting in a dataset comprising four birds only from group 1 on day 14.

### Bacteriology

Transmission of both *S.* Infantis strains from infected to in-contact birds appeared already at seven days of age. Whereas the pESI+ strain could be re-isolated from all birds, except for one, the pESI– strain was detected in 5 of the 15 infected and 4 of the 10 in-contact birds. Interestingly, no statistically significant difference was found in the transmission pattern between both groups (*p* = 1).

**Figure 3.**
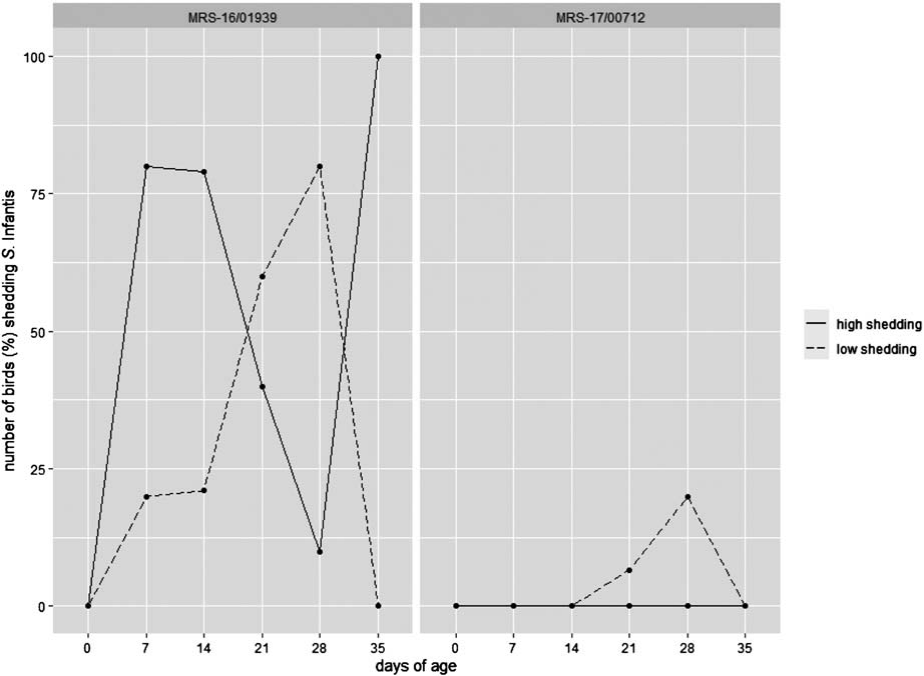
Shedding behaviour of S. Infantis strains MRS-16/01939 and MRS-17/00712 determined by investigation of cloacal swabs.

All cloacal swabs before infection as well as those from birds of the negative control group were negative for *Salmonella* throughout the experiment. Number of re-isolations from birds infected with the pESI+ strain was significantly higher compared to the pESI– strain (*p* = 0.011). Shedding behaviour was also different with the pESI+ strain being re-isolated throughout from all cloacal swabs whereas the pESI– strain was re-isolated only from one and two birds at 21 and 28 days of age, respectively. High shedding was only detected from birds infected with the pESI+ strain, with the highest number of positives at day 7 (80%), day 14 (79%), and day 35 (100%). Re-isolation of the pESI– strain by direct plating did not give any positive results and was only possible following enrichment (Figure 3).

**Figure 4.**
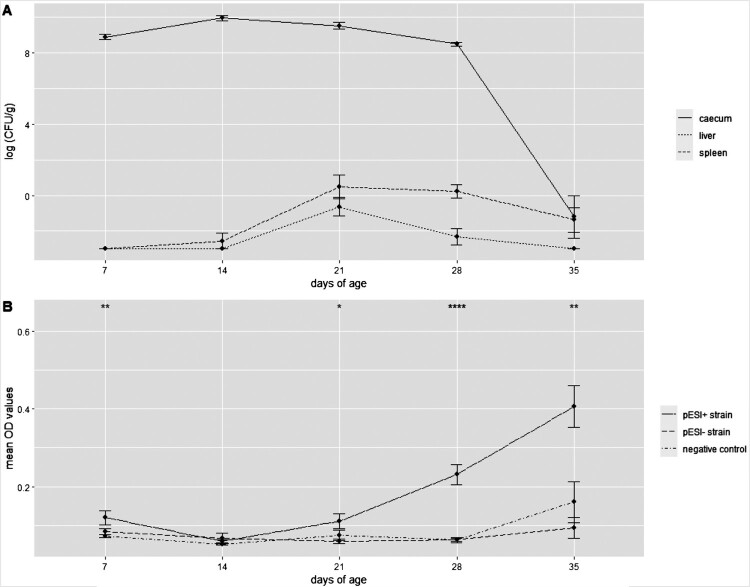
(A) Bacterial load (log (CFU/g)) from organs caecum, liver, and spleen from birds infected with pESI + S. Infantis strain over the time course of the experiment. (B) Homologous antibody response (mean OD values) in birds infected with pESI+ strain, pESI– strain, and negative control birds. Statistically significant differences are presented with asterisks: *p < 0.05; **p < 0.01; ***p < 0.001; ****p < 0.0001.

**Figure 5.**
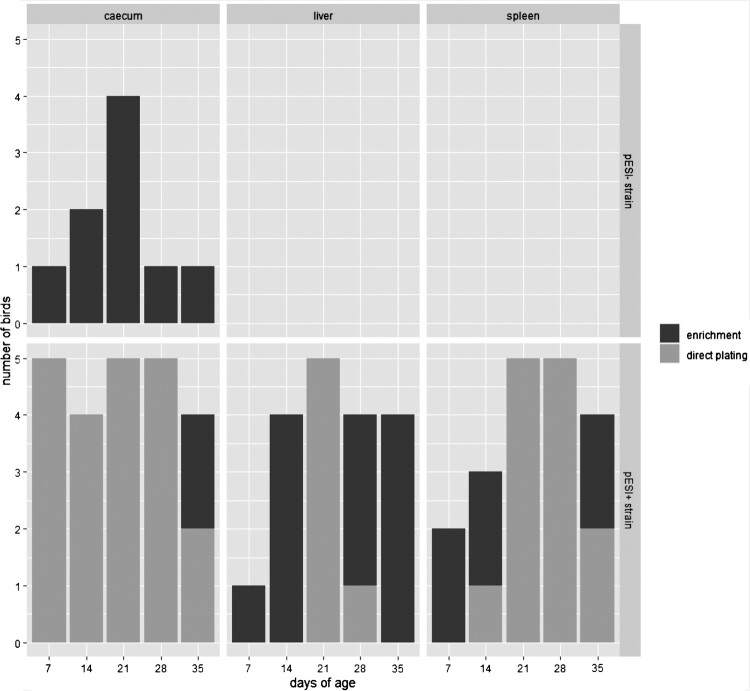
Comparison of direct plating and enrichment from organ samples of birds infected either with pESI + or pESI– strain in regard to the number of birds detected positive. Differences between both strains were statistically significant in caecum (*p* < 0.05), liver (*p* < 0.01), and spleen (*p* < 0.01).

Quantification of *S*. Infantis was only possible for birds infected with the pESI+ strain. Five liver samples from this group were positive by direct plating on day 21 of age (*p* < 0.01) and one liver sample on day 28 with counts ranging from 1 to 7 CFU/g. No positive liver samples were obtained by direct plating at all other dates. Interestingly, spleen samples were positive by direct plating at days 14 (1/4), 21 (5/5; *p* < 0.01), 28 (5/5; *p* < 0.01), and 35 (2/5) with bacterial loads ranging from 1 to 300 CFU/g. The pESI+ strain was re-isolated by direct plating from the majority of caecal samples at all sampling time points: from all birds at the age of 7 (*p* < 0.01), 14 (*p* < 0.05), 21 (*p* < 0.01), and 28 (*p* < 0.01) days, and from one bird at the termination of the study. The bacterial counts ranged from 1 × 10^6^–5 × 10^10^ CFU/g with a clear peak at 14 days of age (Figure 4(A)). Together with enrichment, the number of positive organs from this group resulted in 18 liver, 19 spleen, and 23 caecum samples found positive. In contrast, none of the samples from birds infected with the pESI– strain was positive by direct plating during the experiment. However, on all sampling days, re-isolation of this strain was possible from caecal samples by enrichment. But none of the liver and spleen samples was found positive (Figure 5).

### Serology

No dissimilarities were found in regard to the performance of the two indirect in-house ELISAs. Infection with the pESI+ strain revealed a significant increase of antibody levels in birds at all sampling time points: day 7 of age (*p* < 0.01), day 21 of age (*p* < 0.05), day 28 of age (*p* < 0.0001), and day 35 of age (*p* < 0.01) compared to birds infected with the pESI– strain and negative control group. No difference in antibody levels was found between birds infected with the pESI– strain and the negative control (Figure 4(B)).

## Discussion

Field observations indicate that the ability of *S*. Infantis strains to persist on farms may differ. In Austria, a geographical separation was noticed with strains originating from southern parts of the country persisting heavily on farms in contrast to strains originating from northern parts [13]. Therefore, it can be assumed that the persistence feature of a strain can also be seen as an evidence for an increased or decreased ability to infect and colonize birds. Interestingly, these strains often vary in their phenotype. Besides differences in nutrient utilization, the most outstanding is their diverse antibiotic-resistant profile with MDR of persisting strains [35]. This is of special interest, as in Europe, the proportion of isolates showing MDR in broilers and broiler meat is alarming, accounting for 79% and 75.3% of the MDR *Salmonella* isolates, respectively. This finding is also reflected in human isolates, where actually 41.8% of *S*. Infantis are MDR isolates [36]. Furthermore, various countries report that such MDR strains harbour a pESI-like plasmid containing not only resistance genes but also several virulence factors (15, 17, 19, 37, and 38). *In vitro* studies showed that this plasmid leads to superior biofilm formation, adhesion, and invasion into avian and mammalian host cells [15], which might also explain the persistence characteristic.

So far, experimental infection studies with *S*. Infantis were rare, only based on a single strain and mostly performed in layer-type birds [24, 37, 38]. Therefore, for the first time, an *in vivo* trial was set up in which infectivity features of two different *S*. Infantis strains were evaluated in broilers. Infection via the oral route represents the most important way of horizontal transmission of *Salmonella* in chickens, in the field mainly due to environmental persistence of strains in the stables, resulting in caecal colonization, shedding of the pathogen in faeces, and consistent re-infections of birds [39–41].

In the present study, both strains successfully colonized the intestine but displayed different shedding patterns. Broilers infected with the pESI+ strain could be attributed as high shedders. This strain was already re-isolated from the majority of birds from the first sampling time point until the end of the experiment. Interestingly, the number of high shedders decreased with the course of the experiment until the age of 28 days, rising up again at 35 days of age. This is indicative of an intermittent excretion as reported for *S.* Enteritidis and Typhimurium, which might be caused by the acquisition of competitive microflora or the development of a specific cellular response [42–44]. In contrast, only a few broilers infected with the pESI– strain showed shedding of the strain detected solely when enrichment was applied, reflecting their status as low shedders. The reduced infectivity was also reflected in a delayed excretion, first proved at the age of 21 days. Genetics, housing, age of birds, infection dose/time, and feed are known to influence the frequency and duration of intestinal colonization [39, 44, 45]. As these features were identical in the actual study, the shedding patterns can clearly be attributed to the different nature of the used *S*. Infantis strains. An explanation for this might be the fact that different clones of one *Salmonella* species are able to adapt differently to the chicken intestine, as shown for *S*. Heidelberg in layer chickens [44], or for a large-plasmid-cured variant of *S*. Enteritidis [46]. In the present study, it can be suspected that multiplication of the pESI– strain in the caeca was less effective which resulted in a lower excretion rate of positive birds being under the detection limit of direct plating.

Also, a significant difference in the colonization ability in the caeca was recognized. The pESI+ strain was re-isolated throughout the whole experiment mostly by direct plating resulting in similar data as previously reported from infection/vaccination studies performed in layer-type birds with serovars Infantis and Enteritidis [25]. Furthermore, this strain proved to possess the ability for systemic spread within the broiler birds. Interestingly, re-isolation from the spleen was superior compared to the liver; a finding in clear contrast to previous experimental studies performed in laying hens with other *Salmonella* species in which similar results for spleens and livers were reported [42, 47]. In contrast, the pESI– strain was only detected following enrichment in a limited number of birds, pointing to a reduced ability to propagate or colonize well in the caeca. This strain resided only in the caeca, an observation reported previously after intratracheal application of *S*. Infantis reference strain ATCC^®^51741 in layer-type birds [26]. It can be assumed that this difference could also be attributed to the lack of the pESI-like plasmid, which harbors different virulence genes. For example, *irp2*, *ipf*, and *klf*, are reported to facilitate colonization [35, 48]. But also, the presence of plasmid-encoded fimbrial operons *pef* and *sta* was elucidated, which were shown to mediate adhesion to and invasion in epithelial cells as shown for different *Salmonella* species [49, 50] So far, *S*. Infantis has been presented being less invasive compared to other *Salmonella* serovars [51, 52]. However, based on the present data, this needs to be reconsidered as spreading from the caeca to liver and spleen was demonstrated for the pESI+ strain. A similar feature was shown for *S*. Typhimurium which is able to actively invade host tissues and survive intracellularly if a specific set of virulence factors encoded on the *Salmonella* pathogenicity island 1 and 2 are present [53].

Until 21 days of age, the infection dynamic of the pESI+ strain was characterized by an increase and a peak in the CFU counts. Afterward, a decrease of CFU counts coincided with the rise of antibodies, underlining the development of humoral immunity, similar as reported for other *Salmonella* serovars [54, 55]. In contrast, no antibodies were noticed in birds infected with the pESI– strain throughout the whole experiment. Previous data on *S*. Enteritidis demonstrated that antibody responses largely correlate with the degree of caecal colonization [56]. This finding is also of certain importance for the field, as it indicates possible hindrances in serological screenings for antibodies to reveal *S*. Infantis infections in flocks.

Despite a clear difference in shedding and colonization behavior, both *S*. Infantis strains spread rapidly to in-contact birds, and the respective proportion of positive infected and in-contact birds did not differ. This finding agrees with data from other *Salmonella* serovars [47, 57]. Similar to the situation on farms, the presence of dust together with coprophagia might have contributed to the rapid horizontal spread within groups.

The present study provides for the first time evidence of *S.* Infantis strain-specific virulence in commercial fast-growing broiler birds. An increased potential of *S.* Infantis harboring the pESI-like plasmid to colonize and infect birds was revealed, coinciding with a higher colonization rate in the caeca and the ability to invade inner organs. Awareness needs to be risen to such strains as they have a clear advantage to successfully spread within birds/farms with consequences on transmission to humans. Hence, they act as an emerging public health risk.
